# EPIC Spectral Observations of Variability in Earth’s Global Reflectance

**DOI:** 10.3390/rs10020254

**Published:** 2018

**Authors:** Weidong Yang, Alexander Marshak, Tamás Várnai, Yuri Knyazikhin

**Affiliations:** 1 Goddard Earth Sciences Technology and Research, Universities Space Research Association, Columbia, MD 21046, USA; 2 NASA Goddard Space Flight Center, Greenbelt, MD 20771, USA; 3 Joint Center for Earth System Technology, University of Maryland at Baltimore County, Baltimore, MD 21250, USA; 4 Department of Earth and Environment, Boston University, Boston, MA 02215, USA

**Keywords:** The Earth Polychromatic Imaging Camera (EPIC), sunlit part of the Earth, spectral reflectance, land and ocean, clouds

## Abstract

NASA’s Earth Polychromatic Imaging Camera (EPIC) onboard NOAA’s Deep Space Climate Observatory (DSCOVR) satellite observes the entire sunlit Earth every 65 to 110 min from the Sun–Earth Lagrangian L1 point. This paper presents initial EPIC shortwave spectral observations of the sunlit Earth reflectance and analyses of its diurnal and seasonal variations. The results show that the reflectance depends mostly on (1) the ratio between land and ocean areas exposed to the Sun and (2) cloud spatial and temporal distributions over the sunlit side of Earth. In particular, the paper shows that (a) diurnal variations of the Earth’s reflectance are determined mostly by periodic changes in the land–ocean fraction of its the sunlit side; (b) the daily reflectance displays clear seasonal variations that are significant even without including the contributions from snow and ice in the polar regions (which can enhance daily mean reflectances by up to 2 to 6% in winter and up to 1 to 4% in summer); (c) the seasonal variations of the sunlit Earth reflectance are mostly determined by the latitudinal distribution of oceanic clouds.

## 1. Introduction

The Earth Polychromatic Imaging Camera (EPIC) is a ten-channel earth monitoring spectroradiometer onboard the Deep Space Climate Observatory (DISCOVR) satellite located at Sun–Earth Lagrange-1 (L1) point (http://epic.gsfc.nasa.gov). From this unique location it is able to observe the entire sunlit face of Earth continuously. EPIC has been in operation since June 2015, providing global spectral images of Earth every one to two hours. Together with the National Institute of Standards and Technology Advanced Radiometer (NISTAR) that measures the Earth’s total irradiance in four broadband channels, EPIC provides insights into Earth’s energy balance.

The EPIC camera captures the narrow band spectral images of Earth on a 2048 × 2048 CCD (Charge Coupled Device) array sensor by using a rotating spectral filter wheel inside the EPIC telescope. The ten-channel images include four channels (318, 325, 340 and 388 nm) in the ultra-violet (UV), four channels (443, 551, 680 and 688 nm) in the visible (VIS) and two channels (764 and 780 nm) in the near-infrared (NIR) region. The ten-channel images are used to derive ozone, SO_2_, properties of aerosols, and clouds, as well as properties of vegetated surface such as leaf area index and its sunlit portion [1–6].

The resolution of EPIC images depends on the viewing zenith angle (VZA), and is the highest at the point where the viewing zenith angle is 0°. This point is called the sub satellite point, where a straight line from a satellite to the center of the Earth intersects the Earth’s surface. At this point, the optical resolution of EPIC images is about 10 km, and the instantaneous field of view of a pixel is about 8 km. To reduce the amount of data transmitted from DSCOVR, four pixels are averaged onboard the spacecraft for all bands except the 443 nm band [4,7]. This yields downloaded images of 1024 × 1024 pixels with a sub-satellite optical resolution of approximately 20 km. On the ground, these images are then resampled to match the 2048 × 2048 image size of the 443 nm band [4]. Such images can be used to monitor the motion of clouds and weather systems, diurnal course of vegetation sunlit area, as well as events such as dust storms, biomass burnings, and volcanic eruptions.

Unlike instruments on low-orbit or geostationary satellites, EPIC measures the reflected sunlight simultaneously at all sunlit locations (including polar regions) from sunrise to sunset allowing monitoring seasonal changes in Earth reflectance. Here we take advantage of the unique capabilities of EPIC and study the daily and seasonal variations of global observations that cannot be obtained directly from other instruments.

In this paper, we report on EPIC observations of the global reflectance of the whole globe in individual channels, and on their daily and seasonal variability. We also discuss the mean and the variability of reflectances observed over ocean and land separately, and show their latitude dependence.

These observations from individual channels provide preliminary, yet helpful information to better understand variations of global reflectance and Earth radiation balance. In addition, since the EPIC measures the reflectance from Earth in the nearly backward direction (no shadows are observed), these observations can provide additional information for studying the radiative properties of vegetation surfaces [5]. Furthermore, these observations and analysis provide useful information for studying Earth-like exoplanets [8–12].

The outline of this paper is as follows: In Section 2, we present the data used in our analysis. Then, in Section 3, we discuss our observations of the diurnal and seasonal variations of global reflectance and show how these variations depend on factors such as land–ocean fraction over the sunlit face of Earth, atmospheric molecular scattering, latitude, and cloud distribution. Finally, in Section 4, we summarize the main observations and the conclusions from this work.

## 2. Data and Methods

In this work, we use Level-1B EPIC spectral images spanning from June 2015 to August 2016. For each pixel, the EPIC products provide (1) geo-location (latitude, longitude), solar and viewing zenith and azimuthal angles, and (2) calibrated at sensor reflectance (radiance at the top of the atmosphere (TOA) multiplied by *π* and normalized by the incident spectral solar irradiance). The reflectances are obtained by multiplying the original data values provided in the L1B files in engineering units of count per second by calibration factors for each wavelength (https://eosweb.larc.nasa.gov/project/dscovr/DSCOVR_EPIC_Calibration_Factors_V02.pdf). These calibration factors were obtained by comparing EPIC observations with measurements taken by low Earth orbit satellite instruments [4,7], and analyzing EPIC moon observations [7]. We use the latitude and longitude of pixels to identify the surface types according to the International Geosphere-Biosphere Programme (IGBP) surface ecosystem classifications. In addition, we use the solar zenith angle (SZA) values to select only pixels with SZA < 78°. Pixels with higher SZA values are excluded to avoid complications from the oblique illumination, large field-of-views, and slight variations in the DSCOVR satellite’s position relative to the exact L1 point (orbital data shows that the Solar-Earth-Vehicle angle varies from ~ 4° to ~12° with a mean ~8.4°). It is estimated that the excluded number of pixels is only about 4% of the total number of pixels in sunlit face of Earth, therefore excluding these pixels would not affect the global statistics. The EPIC LIB products and accompanying documentation are available from the NASA Langley Atmospheric Science Data Center (https://eosweb.larc.nasa.gov/project/dscovr/dscovr_table).

In this study, we have considered two different global statistics of EPIC measurements.

The first one, <*R*>_1_, is a simple average of all observed reflectances, defined as

(1) $${\langle R\rangle }_{1}=\frac{\sum_{j=1}^{N}\frac{\pi I_{j}}{F_{0}}}{N}=\frac{\sum_{j=1}^{N}R_{j}}{N}$$

where *N* is the total number of used Earth-viewing pixels, *I_j_* is the radiance observed for pixel *j*, *F*_0_ is the solar spectral irradiance for a plane perpendicular to the incoming solar rays, *R_j_* is the at sensor reflectance reported in the EPIC Level 1B data files for pixel *j*.

We note that <*R*>_1_ is normalized by the solar irradiance, and so its spectral and temporal variations indicate variations in the properties of our planet, and are not affected by temporal changes in Sun–Earth distance or by spectral variations in solar irradiance. This is similar to the case of our current observations of exoplanets and to Cassini observations of Earth (https://www.nasa.gov/mission_pages/cassini/multimedia/pial7171.html). Therefore, <*R*>_1_ can be considered a meaningful average from an astronomer’s perspective.

Global models of climate use surface reflectance to simulate the exchange of fluxes of energy, and mass (e.g., water and CO_2_) between the surface and the planetary boundary layer and how changes in Earth surface properties impact this process. In addition to <*R*>_1_, we also consider statistics of TOA reflectance, <*R*>_2_, defined as

(2) $${\langle R\rangle }_{2}=\frac{\sum_{j=1}^{N}\mu_{j}R_{j}}{\sum_{j=1}^{N}\mu_{j}}$$

where *μ_j_* is the cosine of the viewing zenith angle at pixel *j*.

A rigorous definition of the mean reflectance over the sunlit Earth can be found in [13], and can be expressed as:

(3) $$\langle R\rangle (\Omega )=\frac{1}{\pi }\int_{2\pi }\mu R\chi \text{(}\Omega_{n}\text{,}\Omega_{0}\text{,}\Omega \text{)}d\Omega_{n}$$

where Ω and Ω_0_ are the view and solar direction vectors, Ω_n_ is outward normal to an element on Earth’s spherical surface, μ is the cosine of the viewing zenith angle (μ = Ω · Ω_n_), and *χ* is the indicator function of sunlit points that takes the value 1 if the sensor sees a sunlit element and 0 otherwise. It characterizes mean TOA reflectance per unit of sunlit Earth area. In the backscattering direction, Equation (3) is the geometric albedo [13].

The difference between Equations (1) and (2) is that in Equation (2), pixels are weighted by cosine of the viewing zenith angle. This weighting gives greater weight to the pixels at the center of EPIC images (around “noon” pixels) than to those near the edges (sunrise and sunset pixels).

In this paper, we will present statistics of EPIC’s L1-B reflectance data using both Equations (1) and (2) as an approximation to Equation (3). Throughout the whole paper we define the Earth “reflectance” as the ratio between radiant energy reflected by Earth into direction to the sensor and incident solar irradiance as defined by Equations (1) and (2). We note, however, that calculations using both equations gave only slightly different numerical values and displayed very similar behaviors. Therefore, for convenience, we present only the figures from Equation (2), and provide the statistical values from both methods if their differences are significant.

For each EPIC image, we calculate the global reflectance, the reflectance over land or ocean regions or the reflectance of different latitude regions. To study the daily average reflectance and its variability, we use the arithmetic mean of global reflectances of all images within a day. To reduce sampling biases and uncertainties, we process only those days that have at least five sets of full-spectrum images. The seasonal average reflectance is computed based on the arithmetic mean of daily average global reflectances within a season.

## 3. Results

### 3.1. Global Reflectance

#### 3.1.1. The Daily Variability of Global Reflectance: The Effects of Earth Rotation and Molecular Scattering

**Figure 1.**
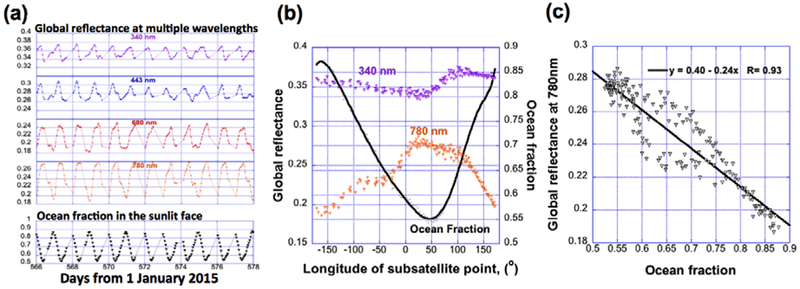
The diurnal variability of global reflectance and its relationship to the ocean fraction over the sunlit face of the Earth from 20 July to 31 July 2016. (Positive longitude means East, negative - West) (**a**) Global reflectance at four wavelengths (340, 443, 680 and 780 nm) and the ocean fraction. (**b**) The ocean fraction and the global reflectance at 340 and 780 nm as a function of the sub-satellite longitude. (**c**) Strong anti-correlation between the 780 nm global reflectance and the ocean fraction. The black straight line is the linear regression fit for the scattered data plot (little triangles) of global reflectance vs. ocean fraction.

In order to understand daily variations in the global reflectance, we use EPIC images acquired over 12 days from 20 July to 31 July 2016 as an example. Figure 1a shows time-series of Earth reflectance at four wavelengths (340, 443, 680 and 780 nm), and the fraction of oceans in the sunlit face of Earth during the same time period. The fraction of oceans here is defined as the ratio of the number of pixels over oceans to the total number of pixels on the sunlit part of the Earth. The global reflectance displays a strong daily cycle that resembles a similar cycle in ocean fraction. This cycle comes from the same areas being illuminated again and again every 24 h. However, the global reflectances behave differently at each wavelength. For example, the noticeable phase difference between 340 nm and 780 nm reflectances is due to the different contribution of surface and atmospheric reflection at these two wavelengths. Since the 780 nm reflectance is mostly from clouds and land surfaces, while the 340 nm reflectance is mainly from clouds and Rayleigh scattering, it is expected that when land occupies the smallest fraction of the Earth’s sunlit face, the 780 nm reflectance reaches its minimum while the 340 nm reflectance does not (as shown in Figure 1a). Figure 1b shows more details of the daily cycles of 340 and 780 nm reflectances as a function of the sub-satellite longitude. The figure shows that 780 nm variations are stronger because of the stronger sensitivity to land area. It also shows that 780 nm reflectance drops only slightly between 30°E and 100°E even though land fraction drops significantly. The eastward increase in 340 nm reflectance over the same area suggests that an eastward increase in cloudiness may play a role keeping 780 nm reflectance high, but an eastward increase in vegetation (having high 780 nm reflectance) may also be a factor. Figure 1c shows the strong anti-correlation between the reflectance at 780 nm and the ocean fractions in the sunlit face. This strong anti-correlation is due to the fact that reflectance at 780 nm is, in addition to clouds, mostly from land surfaces (with minimal contributions from the ocean surface, aerosols, and Rayleigh scattering). For example, when ocean fraction approaches its maximum, the 780 nm reflectance reaches its minimum because the contributions from land surface become small (see Figure 1b). The anti-correlation becomes much weaker at shorter wavelengths due to larger contributions from Rayleigh scattering and to the reduced contrast between land and ocean reflectance.

The above example demonstrates that not only the average values of reflectances, but the ranges of their daily variations also clearly depend on wavelength. Specifically, the daily average reflectances are about 0.35, 0.28, 0.22 and 0.25 (or 0.35, 0.27, 0.21, and 0.23 using <*R*>_1_), and the daily peak-to-peak relative changes are 12, 17, 32 and 41% (or 15, 23, 33 and 44% using <*R*>_1_) of the mean values at each of the four wavelengths. (Note that the daily relative variability would be roughly 3, 4, 9 and 11% (or 3, 4, 8 and 10% using <*R*>_1_), if we used relative standard deviation to represent daily variability). These numbers show that, because of the stronger Rayleigh scattering, the daily average reflectances are higher and the relative variations are lower at shorter wavelengths than at longer wavelengths.

#### 3.1.2. The Daily Average Reflectance and Its Seasonal Variability

**Figure 2.**
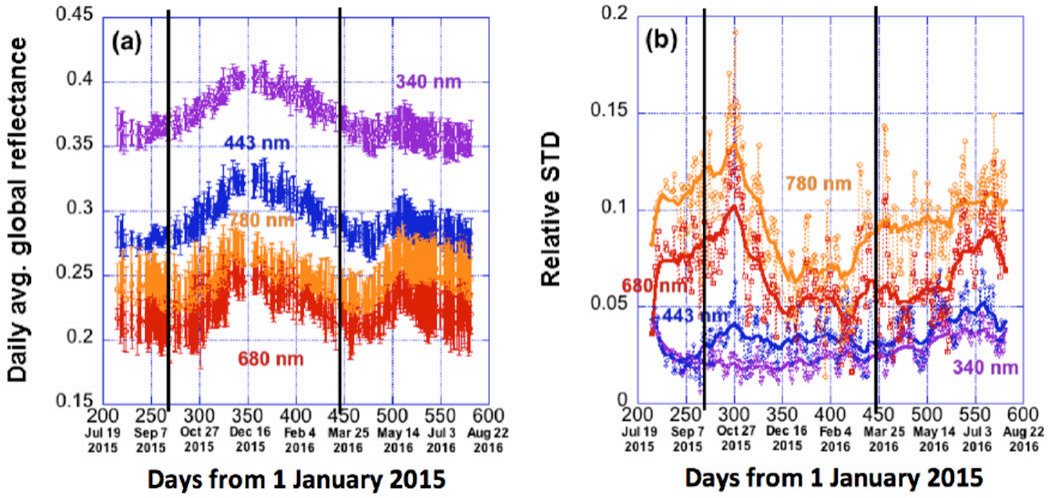
Time-series of daily average global reflectance at four wavelengths (340, 443, 680 and 780 nm) and its variability during a one-year period from 6 August 2015 to 3 August 2016. (**a**) Daily average global reflectance, with error bars indicating the standard deviation (STD) of global reflectance values within each day. (**b**) Relative standard deviation of global reflectance within each day (relative to daily average global reflectance). The solid lines are the smoothed lines of the relative STD data. For the whole year, the relative STDs reach 5%, 6%, 15%, and 18% at the four wavelengths. The two vertical black lines represent the two equinox dates of 23 September 2015 and 20 March 2016.

To study seasonal variations, Figure 2 shows the daily average reflectance and the relative standard deviation values within each day from 6 August 2015 to 3 August 2016. It can be seen that the spectral features described above (higher reflectances and lower diurnal variabilities at shorter wavelengths) remain the same for all seasons through the entire year. However, we also notice clear season-to-season variations during the year: (a) As in Figure 2a, daily average reflectances of all wavelengths show a rise until reaching a winter peak around December and then a decrease for about half year, with another rise from approximately mid-April until reaching a summer peak around the beginning of June. (b) As shown in Figure 2b, relative daily variabilities at longer wavelengths are much smaller between November and March than at other times. This occurs because during this period the sunlit face of Earth is mostly in the southern hemisphere, where the fraction of a more uniform ocean is larger, and the impact of diurnal variations in land fraction is weaker.

The seasonal changes in daily average values are related to seasonal changes in clouds, land, and ocean surfaces. To better understand these changes, we will consider separately the reflections from polar regions, and from land and ocean areas in non-polar regions.

### 3.2. Causes of Seasonal Changes in Daily Average Reflectance

#### 3.2.1. Effects of Polar Regions

The seasonal changes in daily average reflectance described above have included contributions from the bright snow and ice in polar regions. One may wonder whether the seasonal behaviors observed may simply come from the alternating appearances of bright northern and southern polar regions in the EPIC field of view. To see how polar regions affect the Earth’s brightness, we compared the results obtained for the whole globe (discussed above) to the results for latitudes between 60°N and 60°S (which exclude polar regions).

**Figure 3.**
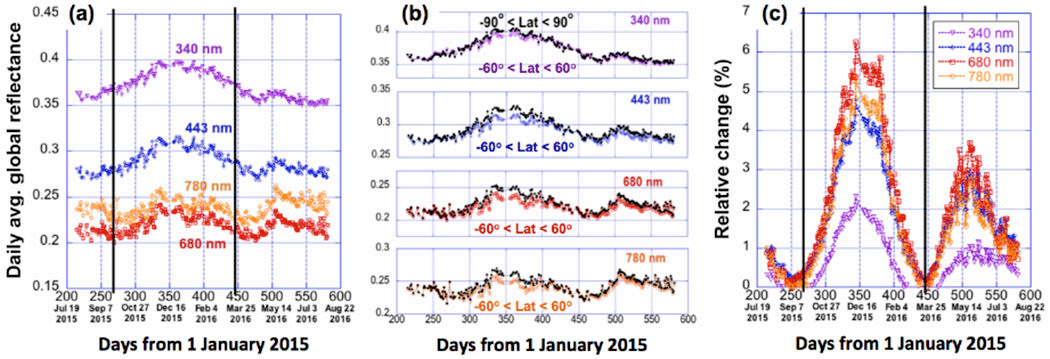
Time-series of daily average global reflectance at different wavelengths. (**a**) Results obtained without polar regions (between 60°N and 60°S); (**b**) channel-by-channel comparisons between results including and excluding polar regions; (**c**) relative impact of polar regions on daily average global reflectance. The two vertical black lines represent the two equinox dates of 23 September 2015 and 20 March 2016.

Figure 3a shows the values of daily average reflectance for latitudes between 60°N and 60°S, while Figure 3b,c show channel-by-channel comparisons of global reflectance with and without the polar regions included. The results indicate that including polar regions increases the daily average reflectance values by 2% for UV and up to 6% for VIS and NIR during winter (due to contributions from Antarctica) and by 1% for UV and up to 4% for longer wavelengths during summer (due to contributions from Arctic). Similar contribution values are also observed using <*R*>_1_ (which shows about the same percentage increase in UV and about 1% more increase for longer wavelengths during winter and summer). This conclusion is very consistent with Jiang et al. [12], who found that Antarctica reflects more sunlight than the Arctic during their respective summers. In addition, excluding polar regions does not change the daily averages during the equinoxes (22 September and 22 March), when polar regions occupy only very small fractions of the EPIC field of view.

These results show clearly that even though the polar regions’ (which are mostly outside the view of geostationary satellites) contributions to the Earth total reflectance are small, they are still significant. However, results in Figure 3 show that polar regions do not alter the overall patterns, and the seasonal behaviors discussed in Section 3.1 are not modified by excluding polar regions. In the following sections, we will be using the latitudes only between 60°N and 60°S for further analysis.

#### 3.2.2. Land and Ocean Regions

In this section, we will subdivide the sunlit face of Earth (without polar regions) into land and ocean regions, and will investigate the daily average land and ocean fractions and the average reflectance values over land and ocean. Here, the land (or ocean) fraction is defined as the ratio of the number of pixels over land (or ocean) to the total number of pixels on the sunlit part of the Earth (SZA < 78° and latitude between 60°N and 60°S).

##### Daily Average of Land and Ocean Regions

**Figure 4.**
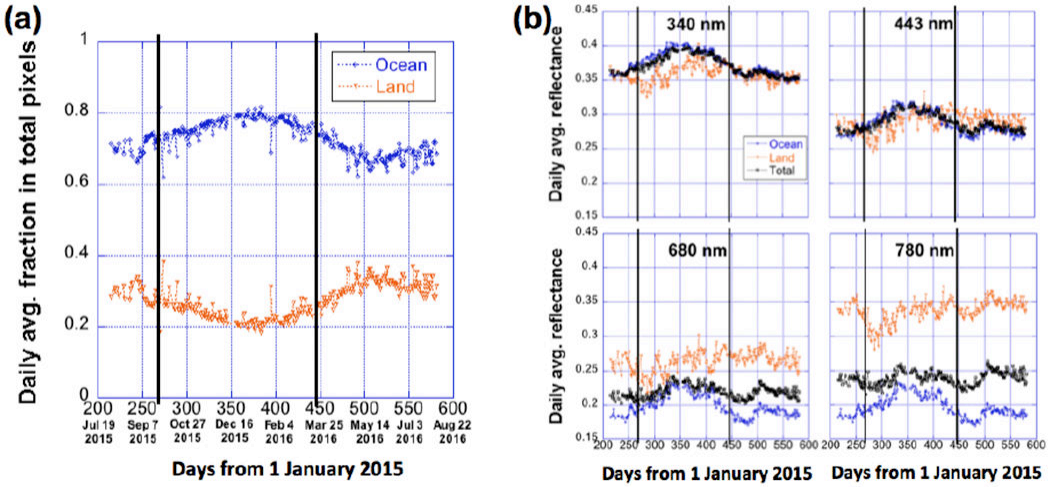
Time-series of (**a**) daily average fractions of land and ocean in sunlit face of Earth, and (**b**) the corresponding daily average reflectance over land and ocean from July 2015 to August 2016. Black curve is the daily average reflectance for the total sunlit face and is used as a reference here. The two vertical black lines represent the two equinox dates of 23 September 2015 and 20 March 2016.

Figure 4a shows the time-series of land fraction, *p*l and ocean fraction, *p*o (with *p*l + *p*o = 1) of the sunlit face of Earth, and Figure 4b shows the corresponding daily average reflectances for land and ocean regions from July 2015 to August 2016. It is noted that the reflectances presented for the land and ocean regions include the contributions from clouds over them.

Figure 4a demonstrates that in all seasons, *p*o is much larger than *p*l, and about 65% (June) to 82% (December) of pixels in the sunlit face of Earth are from ocean regions. *p*o reaches its highest values during the boreal winter, when most of the sunlit areas are in the southern hemisphere, where oceans dominate. Because oceans dominate, one can expect that the seasonal behaviors reported in Section 3.1.2 are determined by ocean regions.

In Figure 4b, the daily average reflectances vary rather differently over oceans (blue curves) than over land (orange curves) at all four wavelengths. This comes from the differences between spectral reflectivities of oceans, land surfaces, and the clouds over them. At 680 and especially at 780 nm, ocean water is generally much darker than land and reflectance is less affected by air molecules and aerosols [14], and so the daily averages are much higher over land (orange curves) than over ocean (blue curves). At 340 and 443 nm, reflection from land and ocean surfaces are weak (except from the icy polar regions) and can be overwhelmed by reflection from clouds, atmospheric aerosols, and air molecules [15–17], and since there are more clouds over ocean than over land [18], the daily average reflectances are slightly lower over land than over ocean.

Finally, Figure 4 shows clearly that, as *p*o is much higher than *p*l, the global reflectance values (black curve) are much closer to those over ocean than to those over land.

##### Northern and Southern Oceans

Since seasonal patterns of daily average reflectances are mostly determined by ocean, our next focus is on oceans on the northern and the southern hemispheres. Here we define the daily average fractions of the total ocean as *p*o,n for northern oceans and *p*o,s for southern oceans (*p*o,n + *p*o,s = 1).

**Figure 5.**
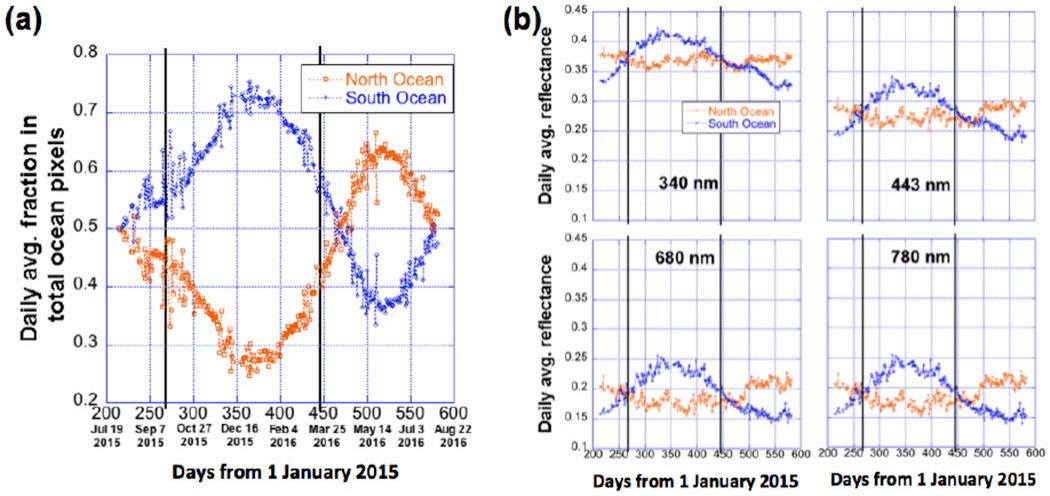
Time-series of (**a**) daily average fraction of northern and southern oceans within the total sunlit oceans and (**b**) daily average reflectance over northern (orange) and southern (blue) oceans from July 2015 to August 2016. The two vertical black lines represent the two equinox dates of 23 September 2015 and 20 March 2016.

Figure 5a shows that during the time period from August 2015 to April 2016, when most of the sunlit face is covered by southern oceans, *p*o,n < *p*o,s and that from May to July, *p*o,n > *p*o,s. Thus, daily average reflectance (Figure 4b) is dominated by southern oceans from fall to spring and by northern oceans at winter.

Figure 5b illustrates the daily average reflectance of northern and southern oceans at four wavelengths. In addition to the spectral behaviors due to Rayleigh effects discussed earlier, it also shows that patterns are very different over the northern and southern oceans. In essence, the daily average reflectance of southern oceans shows distinctive seasonal variations at all wavelengths (blue curves), rising in boreal winter and falling afterwards, while the variations of the reflectance over the northern oceans are much weaker (brown curves).

Why are the patterns of southern and northern oceans so different? Assuming that in all ocean regions reflection from surface and scattering from air molecules and aerosols are relatively uniform both spatially and temporally, the difference in patterns most likely comes from different cloud contributions over the northern and southern oceans. To this end, we recall the results of King et al. [18] on the seasonal variations of cloud spatial distributions based on more than 12 years of MODIS data. MODIS’s results indicate that cloud properties (e.g., cloud fraction and optical thickness) are different for different latitudes and seasons.

##### Latitude-Dependence of Daily Average Reflectance over Oceans

**Figure 6.**
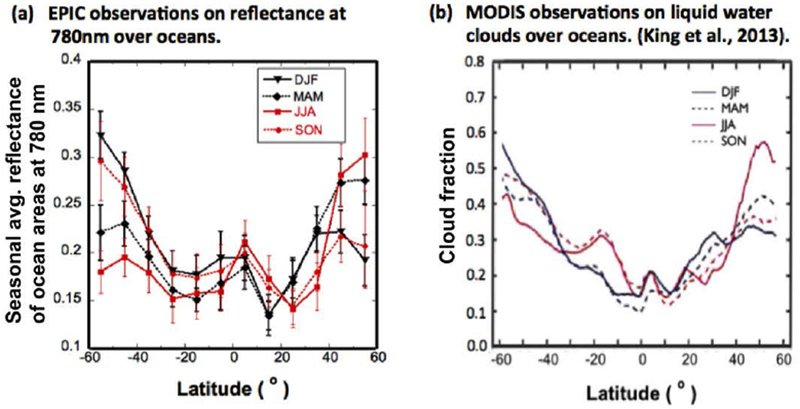
(**a**) The latitude distribution of EPIC’s seasonal average reflectance over oceans at 780 nm, and (**b**) the latitude distribution of MODIS’s water cloud fraction for four seasons. (**b**) Is from King et al. [18] with permission from IEEE Transactions on Geoscience and Remote Sensing.

To examine the impact of clouds on the seasonal variations of daily average reflectance over oceans, here, we separate the northern and southern oceans into twelve 10° wide latitude bins between 60°N and 60°S, and compare the latitude distribution of the seasonal mean daily average reflectances at 780 nm (Figure 6a) with the distribution of water cloud coverage in MODIS observations (Figure 6b) over the four seasons. We focus on 780 nm reflectances because they are much more sensitive to clouds than to the ocean surface.

Figure 6a shows that in boreal winter, the reflectance values at high latitudes of Southern oceans (solid black curve) are larger than those at other latitudes, while in boreal summer, the reflectance values of northern oceans (solid red curve) are larger at high latitudes than at other latitudes. Additionally, the reflectance values at high latitudes of Northern oceans in boreal summer decrease more than Southern ocean reflectances do in the austral summer (in boreal summer, northern reflectance values drop by 44% between 55°N and 35°N, while in austral summer, the values drop by 31% between 55°S and 35°S). Since over oceans, the signal at 780 nm comes from clouds, the features in Figure 6a reflect the distribution of clouds, and are found to be consistent with the latitude distributions of cloud coverage shown in King et al. [18] (Figure 6b).

Naturally, the observations of Figure 6a are related to the overall distribution of cloud reflectivity and are also including the contributions from ice clouds. However, the cloud fraction of water clouds (as in Figure 6b) seems to be a key factor, while the contributions from ice clouds (many of which are weakly reflecting cirrus clouds) and variations of water cloud reflectivity appear relatively less significant. Details of the relationships between the reflectances in Figure 6a and the major contributors are worth deeper investigation. Nevertheless, the consistencies between Figure 6a and Figure 6b indicate that Figure 6a reflects the variations in the latitudinal and seasonal distributions of clouds.

These observations not only provide the information of the radiative contribution of the oceanic clouds at different latitudes, but also reveal the reason why the reflectances from Northern and Southern ocean are different, as illustrated in Figure 5b, and they even explain the variability of the global reflectance as in Figure 3a. First, in boreal winter when most of the Southern oceans face the sun, EPIC sees more bright clouds at high latitudes, thus the reflectance of Southern oceans is larger in boreal winter than in other seasons. Similarly, in summer when most of Northern oceans face towards the sun, EPIC observes more bright clouds at high latitudes of Northern oceans, making the reflectance of Northern oceans larger in summer than in other seasons (Figure 5b). Second, since oceans dominate over land (Figure 4a) and Southern oceans dominate over Northern oceans in winter (similarly, Northern oceans dominate in summer) (Figure 5a), the winter peak and the summer peak in the global reflectance (Figure 3a) are mostly from the bright clouds of high latitudes over Southern oceans in the winter and over Northern oceans in the summer, respectively. Third, due to the decrease of the reflectance with latitude over Southern oceans in boreal winter is slower than that over Northern oceans in summer, EPIC sees more bright clouds from Southern oceans in boreal winter than over Northern oceans in summer. Therefore the summer peak in the global reflectance is lower than the winter peak (Figure 3a).

We note, however, that the curves in Figure 6a,b may not be comparable due to the fact that while MODIS observations are taken near local noon, EPIC observations span the whole day from sunrise to sunset. Indeed, this difference can be important. However, the effects of such differences are small and do not change the statistics on latitude-dependence of EPIC reflectances or MODIS cloud fractions. This is because having fewer data samples at high SZA greatly reduces the weights of radiative contributions of high SZAs, e.g., from the morning and afternoon areas in the EPIC field of view. In effect, the reflectance statistics is mostly contributed from the central Earth region (around noon hours, with low SZA), which makes the reflectance statistics of EPIC more comparable to MODIS in terms of measurement time. Moreover, results using Equation (1) did not show noticeable differences from Figure 6a, which indicates that averaging of the morning and afternoon clouds would generate similar statistics as using noon data.

Nevertheless, it can be concluded that since oceans dominate (Figure 4a), clouds over oceans strongly affect the shortwave reflectance over the whole globe. Southern oceans dominate in winter while Northern oceans dominate in summer. The variability of reflectivity from Southern and Northern oceans is very different (Figure 5b). The transition of dominancy from Southern to Northern oceans happens in April (Figure 5a); this explains that the minimum reflectance for all wavelengths also happens in April.

## 4. Summary

As an initial study of the radiative properties of Earth’s surface and atmosphere observed from the unique position of the DSCOVR satellite, this work studies spectral reflectance of sunlit side of earth using observations taken by the EPIC instrument.

We first characterize the diurnal variability of global reflectance in a 12-day-long dataset. Compared to longer wavelengths, the shorter wavelengths show higher daily mean and lower variability mostly due to stronger molecular and aerosol scattering. At four wavelengths (340, 443, 680 and 780 nm), the daily mean are 0.35, 0.28, 0.22 and 0.25, while the daily maximum to minimum variability are 12, 17, 32 and 41% of the mean values, respectively. Furthermore, the global reflectances at all channels show a cycle of 24 h [10], which, at least at the longer wavelengths, resembles a structure of ocean fraction over the sunlit face of Earth (Figure 1a).

The daily averages of global reflectances display seasonal variations, with a larger peak during boreal winter, and a smaller peak during boreal summer at all wavelengths. For the whole year, the relative standard deviations of daily average reflectance values are 5, 6, 11, and 18% at the four wavelengths (340, 443, 680 and 780 nm), respectfully.

We then analyzed what factors contribute the most to these seasonal variations. First, it was found that the seasonal variations do not change substantially even when the bright polar regions are not included. When the polar regions are included, the global reflectivity increases by 2–6% in winter (due to Antarctica) and 1–4% in summer (due to Arctic) relative to the values around equinoxes (the increases are weakest in UV channels).

We then separated the sunlit side of Earth into land and ocean areas. It was found (Figure 4) that the seasonal variations are mostly determined by ocean areas, simply because the daily average fraction of oceans over the sunlit side of the Earth is always much larger (65–80% depending on season; with maximum during boreal winter) than the fraction of land. It is also found that the variations over oceans are dominated in August–April by southern hemisphere and in April–August from northern hemispheres; they follow very different patterns (Figure 5b).

To understand the cloud effects on the northern and southern ocean reflectance, we analyzed the latitude dependence of 780 nm reflectances over oceans. Results showed that in boreal winter, reflectances of Southern oceans are larger at the high latitudes than at other latitudes, while in summer reflectances of Northern oceans are larger at the high latitudes than at other latitudes. This latitude dependence is consistent with MODIS data on cloud coverage, indicating that cloud coverage is a key factor governing the seasonal variations observed by EPIC.

Overall, EPIC observations show that while contributions from clouds are significant and important, the diurnal variations of reflectance are strongly affected by changes in the land–ocean ratio. The seasonal variations of reflectance, however, are mostly determined by the distribution of clouds. These results illustrate that studying the variations of spectral reflectances using EPIC data can provide valuable insights into the radiative properties of the Earth’s sunlit side and into their relationships to the surface and atmospheric properties.
